# Harvard University

**Published:** 1893-12

**Authors:** 


HARVARD UNIVERSITY.—(SCHOOL OF VETERINARY MEDICINE).

*June —.—Degree M. D. V.*

| | |
|---|---|
| Cronon, Patrick James | Canton, Mass. |
| Dowd, Eugene Anthony | Roxbury, Mass. |
| Naylor, William Alfred | Dorchester, Mass. |
| Lambert, Horace D. | (?) |

